# Piezoelectric Films Based on Polyethylene Modified by Aluminosilicate Filler

**DOI:** 10.3390/polym11081345

**Published:** 2019-08-13

**Authors:** Halina Kaczmarek, Bogusław Królikowski, Marta Chylińska, Ewa Klimiec, Dagmara Bajer

**Affiliations:** 1 Faculty of Chemistry, Nicolaus Copernicus University in Toruń, ul. Gagarina 7, 87-100 Toruń, Poland; 2 Łukasiewicz Research Network—Institute for Engineering of Polymer Materials and Dyes–Toruń Division, ul. M. Skłodowskiej-Curie 55, 87-100 Toruń, Poland; 3 Institute of Electron Technology—Kraków Division, ul. Zabłocie 39, 30-701 Kraków, Poland

**Keywords:** poly(ethylene), polymer composites, aluminosilicates, piezoelectric properties, composite characterisation

## Abstract

The development, universality and miniaturization of electronic devices leads to the search for new piezoelectric materials, among which recently, polymers play an increasingly important role. In this work, composites based on two types of polyethylene—high density polyethylene (HDPE), and medium density polyethylene (MDPE)—and aluminosilicate fillers were obtained by extrusion process. This method allowed obtaining flexible electrets in the form of a thin film after polarization under a constant electric field of 100 V/μm. The morphology of the composites was characterized by scanning electron microscopy, whereas the crystallinity was determined by X-ray diffraction. The mechanical properties and thermal stability of the composites were examined by means of tensile tests and thermogravimetry, respectively. The piezoelectric characteristics were appointed by measuring the electric charge and the voltage in the polarized samples. Piezoelectric coefficients, and the stability of electrets over time were also determined. Moreover, the effect of film orientation on piezoelectric properties was investigated. Composites with appropriate morphology (i.e., well dispersed filler particles in the polymer matrix and formed holes) were obtained which ensured permanent electrical polarization. It was found that the best piezoelectric, mechanical properties and thermal stability exhibits HDPE composite with 5% of aluminosilicate filler.

## 1. Introduction

The rapid development of microelectronics is connected with the exploration of new smart or intelligent materials, some of which include piezoelectrics, for example. These materials are characterized by reversible response to mechanical stress or electric stimulus. In other words, they generate an electric charge under the influence of mechanical force or they are deformed in the electric field. Although the piezoelectric phenomenon has been described in many works and monographs [1,2,3,4,5] (and references cited therein), the dependence of piezoelectric properties on the material structure is still not well understood.

The first piezoelectric materials were based on inorganic compounds, mainly of the perovskite type ceramics (zirconate-titanate, PZT), and did not belong to environmentally friendly compounds due to the presence of heavy metals (e.g., Ba, Pb). They also had limitations related to high brittleness, rigidity and very high processing temperature that hindered the formation of any shape. Thanks to their good piezoelectric properties, they are still used today, mainly in microelectronics, photovoltaics and sensor production. Currently, however, new materials with a strictly planned nanostructure (nanowires, nanorods, nanotubes) are being sought, that can meet the requirements of modern technologies [6,7,8,9,10,11]. Molecular solid solutions i.e., hybrid inorganic–organic systems, which combine the properties of both groups of compounds, are examples of such novel piezoelectrics. 

As was recently reported, the new class of organic ferroelectrics, with a large piezoelectric response, even higher than that in lead zirconate titanate, was developed [6,7]. These were halogen-containing crystalline organic salts: trimethylchloromethylammonium (TMCM) or trimethylfluoromethylammonium (TMFM) with MnCl_3_ or CdCl_3_. Using CdBr_3_ instead of tribromocadmium(II) led to specific halogen-bonds (i.e., interactions between Cl-cations and anionic Br atoms) forming nucleofilic and electrophilic regions [8]. Such structure leads to breaking crystal symmetry and spontaneous permanent polarization guaranteeing high values of piezoelectric parameters. 

Promising solutions in the design of piezoelectrics are materials containing carbon nanotubes (CNT) [9]. In this work, piezoelectric composite was formed in the process of direct growth of PZT on multi-walled CNTs and dispersed in the polytetrafluoroethylene (PTFE) matrix. Such a procedure enabled obtaining a significant increase of the output voltage by an external mechanical force. PTFE played the role of a binder and provided material flexibility. 

In addition to advanced experimental research leading to the development of new materials with expected properties, there are high hopes of the use of supporting theoretical studies. An example is the use of calculations, in particular, the double asymptotic homogenization method for the prediction of the best composition of 1–3 type cement-based piezoelectric composites (with different PZT fraction). Good agreement between theoretical calculations and experimental data was found, which confirms the correctness of the model used [10].

Besides inorganic substances and biological systems, synthetic semi-crystalline polymers also exhibit such properties. They have been found to have a wide range of uses due to their numerous advantages such as high flexibility, low production cost, ease of processing, possibility of chemical modification, ability to form various shapes and stability [11,12,13,14,15,16]. 

The piezoelectric polymers, among which fluoropolymers such as poly(vinylidene fluoride) and its copolymers play an important role [17,18,19,20,21], already have a variety of applications, e.g., for the production of sensors, transducers and energy harvesters. There is also a huge demand for such materials in medicine and biology [22,23,24]. Examples of original applications are personal sensors that use human work in motion, and even the work of the heart or the lungs with every breath. Another aspect that should be emphasized is the search for ecological, alternative energy sources using devices based on piezoelectrics [25,26].

Recently, an interesting work on PVDF electrospun mats modified by silver nanoparticles (Ag-NPs) was published [27]. This system was characterized not only by good piezoelectric properties but also by good mechanical strength and thermal stability at small content of 0.4–0.6% Ag-NPs despite the weak component adhesion. The effect of the Ag-NPs addition was higher β-phase content of PVDF (even up to about 8%), which is responsible for the piezoelectric properties of this polymer, and consequently, dielectric permittivity and interfacial polarization also increased. 

Another example of a modern hybrid material is a composite containing magnetoelectric alloy (Terfenol-D composed of Tb, Dy and Fe), CoFe_2_O_4_ and poly(vinylidene-trifluoroethylene) matrix [28]. The undoubted advantage of this system was the high piezoelectric response, regardless of the ratio of inorganic fillers.

The high expectations are related to polyolefins, popular semi-crystalline polymers used for large-scale production. There are many papers describing the piezoelectric properties of isotactic polypropylene [29,30,31,32,33], however, polyethylene (PE), despite its many advantages, has not been used as a piezoelectric in practice so far. Our recent studies have shown that PE, after appropriate modification, can also be considered as a potential piezoelectric material [34]. One of the ways to obtain piezoelectrics is by foaming the polymer, and the other is the addition of inorganic fillers. Both methods lead to the formation of a cellular, porous structure in which an electric charge can accumulate.

In this article we present the new results concerning composites based on two types of polyethylene filled by aluminosilicate, which were used for easy creation of flexible, stable electrets. In order to ensure the practical use of the proposed new materials, they must be carefully characterised, in particular their morphology, degree of crystallinity, mechanical strength and thermal stability have to be determined. Moreover, the conditions of their fabrication have to be carefully planned and strictly maintained to obtain repeatable properties.

## 2. Materials and Methods

### 2.1. Materials

Two types of PE were used for composite preparation: high density polyethylene (HDPE with a density of 0.946 g/cm^3^) Tipelin FS 471-02, and medium density polyethylene (MDPE with a density of 0.938 g/cm^3^) Tipelin FS 383-03, both produced by MOL Petrochemicals Co., Ltd., Hungary [35,36]. The inorganic filler was Sillikolloid P87 (Hoffmann Mineral GmbH, Germany) containing 80% SiO_2_, 14% Al_2_O_3_, 1% Fe_2_O_3_ and 5% other minerals (in this amorphous phase is 10%) [37]. 

### 2.2. Composite Preparation

The filler was added to the polymer matrix in an amount of 2.5, 5 and 10 wt.%. Components (polymer and filler) were mixed at room temperature and then extruded in a co-rotating twin-screw extruder of type BTSK 20/40D (Bühler GmbH, Braunschweig, Germany) at 185–195 °C to obtain polymer composite in a pelletized form. The pellets were extruded using single-screw extruder PLV 151 (Brabender OHG, Duisburg, Germany) at 225–235 °C to obtain a cast film of width ca. 140 mm. The film samples were then oriented in a ratio of 3:1 in the uniaxial stretching device executed by Institute for Engineering of Polymer Materials and Dyes, Toruń, Poland. This was a two-stage process. In the first stage, the film was heated up and oriented at 120–105 °C; in the second stage, the film was cooled down to 90–80 °C. The thickness of the non-oriented and oriented film samples was 112–126 µm and 63–89 µm, respectively.

### 2.3. Characterization of Composite Properties

Surface morphology of the samples was observed using scanning electron microscopy (SEM 1430 VP, LEO Electron Microscopy Ltd., Cambridge, UK) combined with an X-ray spectrometer (EDX) Quantax 200 with an XFlash 4010 detector from Bruker AXS, Karlsruhe, Germany. Brittle breakthroughs were made in liquid nitrogen to visualize the interior of the composites. The samples for SEM images were sputter coated with gold.

X-ray diffraction (XRD) was done using X’PERT Pro Philips Diffractometer MPD (Ni-filtered Cu K_α1_ radiation, wavelength 1.54056 Å, PANalytical B.V., Almelo, The Netherlands). Range of 2θ measurements was 10–90°, step size 0.02° and time per step 3 s. For calculation of crystallinity degree, the XRD pattern in diffraction angle range of 14–28° was deconvoluted. The strong peaks attributed to crystal phase were fitted using Voigt function, whereas the broad amorphous peak was better adjusted with the mixed Gaussian-Lorentzian function. The straight line was applied as a background. The degree of crystallinity (*X*, %) is the ratio of the area under the peaks corresponding to the signals of the crystalline phase to the total area under XRD (which is the sum of the crystalline reflections and the amorphous halo). The surface area of signals from Sillikolloid were subtracted to avoid overestimation of the polymer crystallinity.

Mechanical properties were determined with the help of TIRAtest 27025 (TIRA Maschinenbau GmbH, Schalkau, Germany) apparatus with the feed speed of crosshead 1.0 mm/min (at the beginning of measurement i.e., in the range of 0–2% extension, to accurately determine the Young’s modulus) and then at 100.0 mm/min until the sample was broken. The dimensions of the measurement section were 50 mm (length) × 15 mm (width). The results were an average value of at least 6 measurements. The error ranges (standard deviations, σ and coefficient of variation, CV) for individual mechanical parameters were: σ = 1.5–5.4 MPa and CV = 5.7–10.7 for σ_M_ (maximal stress); σ = 1.5–7.3 MPa and CV = 5.7–13.2 for σ_B_ (stress at break); σ = 1.6–25.4% and CV = 3.7–9.5 for ε_M_ (maximal elongation); σ = 11.1–25.5% and CV = 3.7–13.7 for ε_B_ (elongation at break); σ = 12.1–24.8 MPa and CV = 2.9–4.7 for *E*_t_ (Young modulus).

Thermal stability was studied using simultaneous TGA-DTA Thermal Analysis TA Instruments type SDT 2960 (New Castle, DE, USA) in nitrogen atmosphere and at heating rate of 10 °C/min in the range from room temperature to 700 °C. The device allows simultaneous recording of TG/DTG and DTA curves. The mass of the sample was a few milligrams.

FTIR spectra were recorded using Vertex 70v (Bruker Optics GmbH, Ettlingen, Germany) with RT-DLaTGS wide range detector and attenuated total reflectance (ATR) mode (with diamond crystal).

Piezoelectric properties of filled PE samples were induced under constant electric field (100 V/μm) at 85 °C during 1 h. The density of piezoelectric charge (*q*) and voltage (*U*) were measured under the stress (*P*) of up to 100 kPa using an electromagnetic actuator (ITE, Cracow, Poland), Arbitrary Waveform Generator Tektronix AWG420 (Electronic Test Equipment, Cary, NC, USA), P334 power amplifier (Meratronik, Warsaw, Poland), tensometric force sensor XFL212R (Measurement Specialties, Inc., international company), ADR 154 amplifier (FGP Sensors Inc., France), electrometer Keithley 6517A with resolution 1 pC (Keithley Instruments, Cleveland, OH, USA), and oscilloscope LeCroy LT-341 (LeCroy, Chestnut Ridge, NY, USA). The surface of contact electrodes was 10 cm^2^. To check the durability of the electrets, the measurements were repeated systematically for samples stored up to 2–3 months. The piezoelectric coefficients, d_33_ and g_33_, were calculated from the relations *q* = f(*P*) and *U* = f(*P*), respectively [29,31,33]. 

## 3. Results and Discussion

### 3.1. Morphology of Polyethylene Composites—SEM/EDX Studies

**Figure 1:**
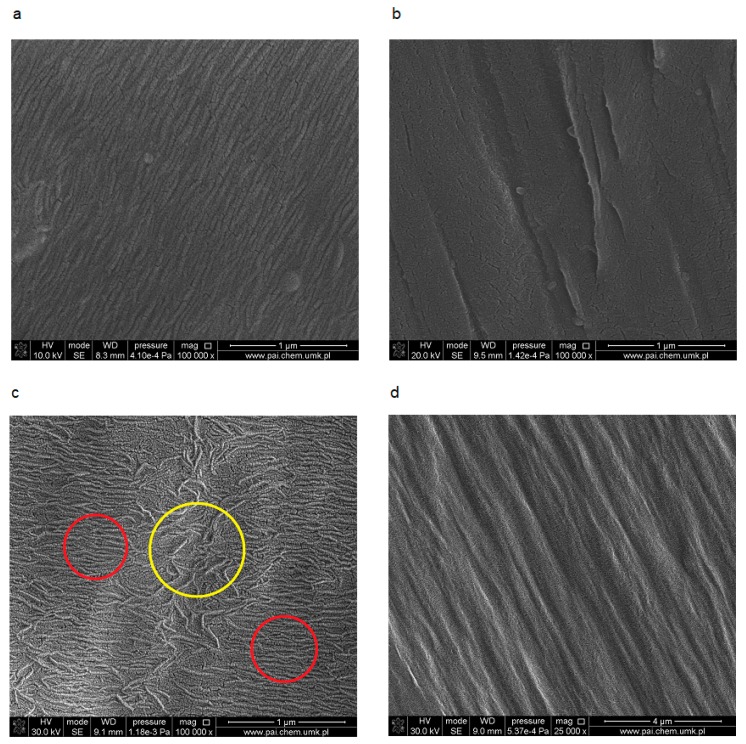
SEM images of HDPE (**a**,**b**) and MDPE (**c**,**d**): Non-oriented (on the left), oriented 3:1 (on the right); red circles indicate the domains with parallel packed fibrils, yellow circle shows entangled fibrous area.

The SEM imaging of neat, non-oriented PE sample surfaces (both HDPE and MDPE) shows the presence of parallel stripes typical for ordered phase (Figure 1a–d), which consist of regularly folded lamellae. Compact, dense packing of ordered structures is clearly visible in SEM images of HDPE (Figure 1a). A similar surface structure in PE was observed by other authors using AFM tapping mode [38].

SEM of MDPE shows the regions with larger and smaller order (they are marked by circles in Figure 1c). The orientation of the samples led to the changes in the structure in both cases. The small corrugations disappear, this may suggest the formation of thicker fibrils (Figure 1b,d). Moreover, during stretching accompanied by deformation of the material, the cavitation process leading to microporosity took place [39].

**Figure 2:**
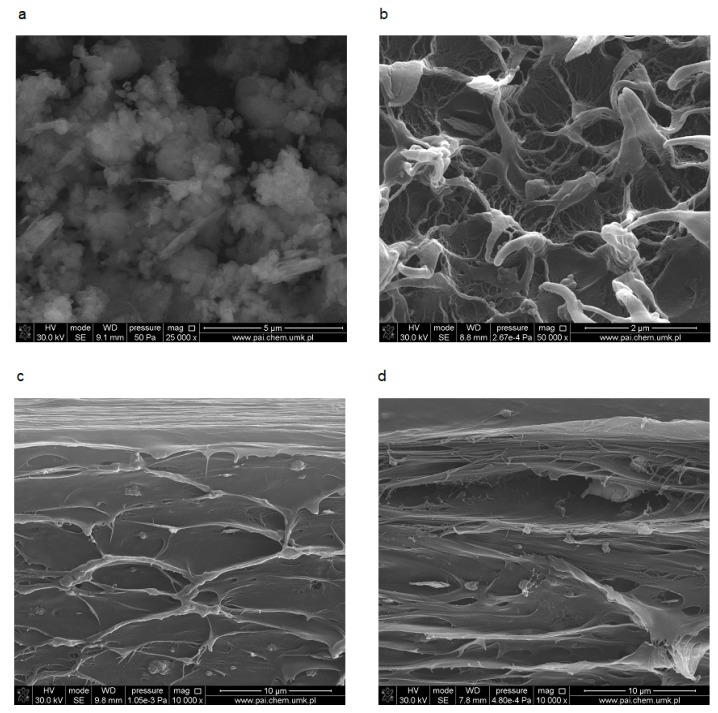
SEM images of Sillikolloid P87 (**a**), MDPE + 5% Sillikolloid P87 non-oriented (NO) (**b**), MDPE + 5% Sillikolloid P87 oriented (O) (**c**), MDPE + 10% Sillikolloid P87 oriented (O) (**d**); c–d, cross-sections.

The morphology of applied filler is presented in Figure 2a. Sillikolloid P87 is a mixture of particles with different shapes, varying degrees of fineness and aggregation. This is a result of its complex mineral composition, i.e., from the presence of both crystalline and amorphous silica particles in the vicinity of which there are also other oxides (Al_2_O_3_, Fe_2_O_3_). There are more or less regular spherical particles and lamellar plates.

An example of the cross-section of MDPE modified by Sillikolloid P87 composite is shown in Figure 2b. In non-oriented samples with Sillikolloid P87, irregular filler particles are surrounded by a polymer and cavities are formed simultaneously. During the orientation process, these voids in the polymer matrix are stretched and enlarged, taking more ellipsoidal shapes (Figure 2c,d). It is connected with irreversible deformation of macromolecules in amorphous phase [39]. 

**Figure 3:**
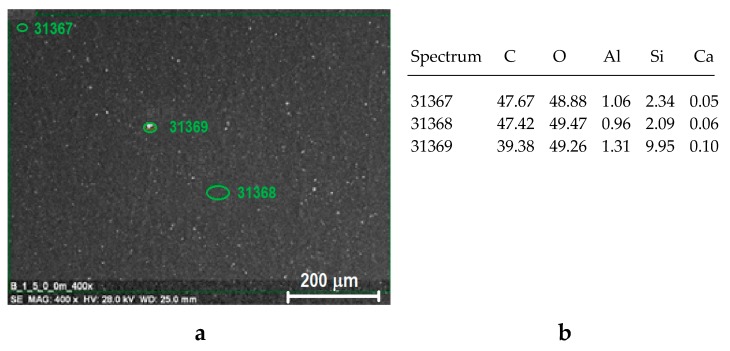

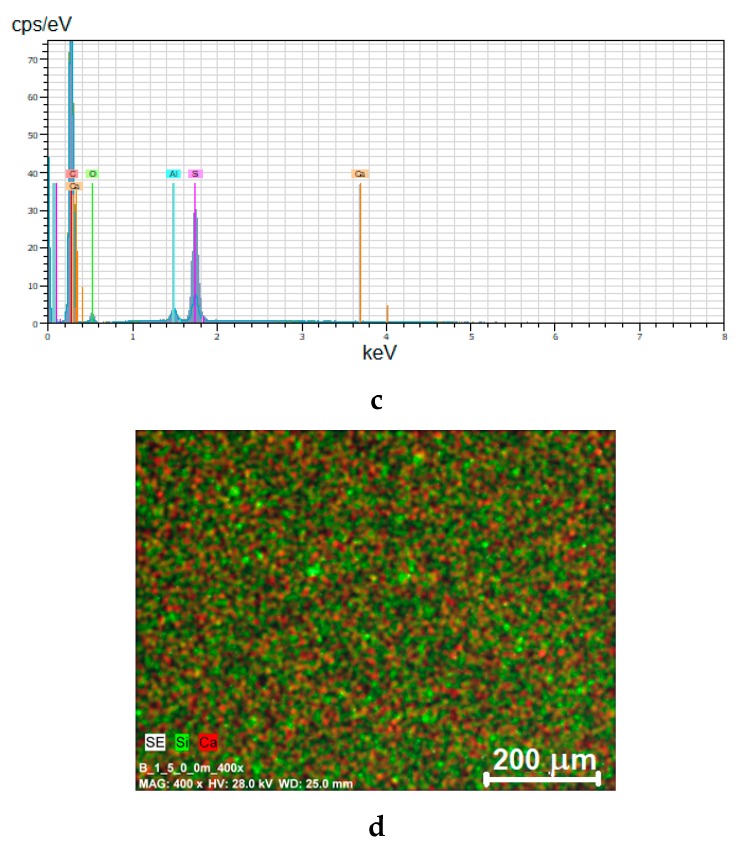
Results of SEM/EDX analysis for non-oriented (NO) sample of HDPE + 5% Sillikolloid P87; SEM image showing EDX analysis points (**a**), elemental composition in the points indicated in Figure 3a (**b**), EDX spectrum (**c**), mapping showing distribution of two selected elements (Si, Ca) (**d**).

From the point of view of the properties of composites, not only the type and ratio of components is important, but also the degree of dispersion of the additive in the polymer matrix. In order to check the degree of dispersion of fillers in the polymer, EDX analysis was carried out, which allows for the detection of individual elements occurring in the chemical structure of the components of the composite, and the observation of their distribution on the surface of the sample (i.e., so-called ‘element mapping’). This analysis showed a homogeneous distribution of elements on the surface of the samples, regardless of the type of polymer as well as the components ratio. Also, the orientation does not significantly affect the change in the distribution of elements. An example of EDX results for HDPE with 5% Sillikolloid P87 with the distribution of the most important elements occurring in the samples, i.e., C, O, Si, Ca and Al is shown in Figure 3. 

Due to the different nature of PE (hydrophobic) and filler (hydrophilic), there are no specific interactions between the components of the composite. However, even the lack of polymer-filler adhesion or very weak interfacial interactions do not exclude the improvement of the composite properties, including piezoelectric properties, as exemplified by the work by Issa et al. [27].

### 3.2. X-ray Diffraction Analysis

**Figure 4:**
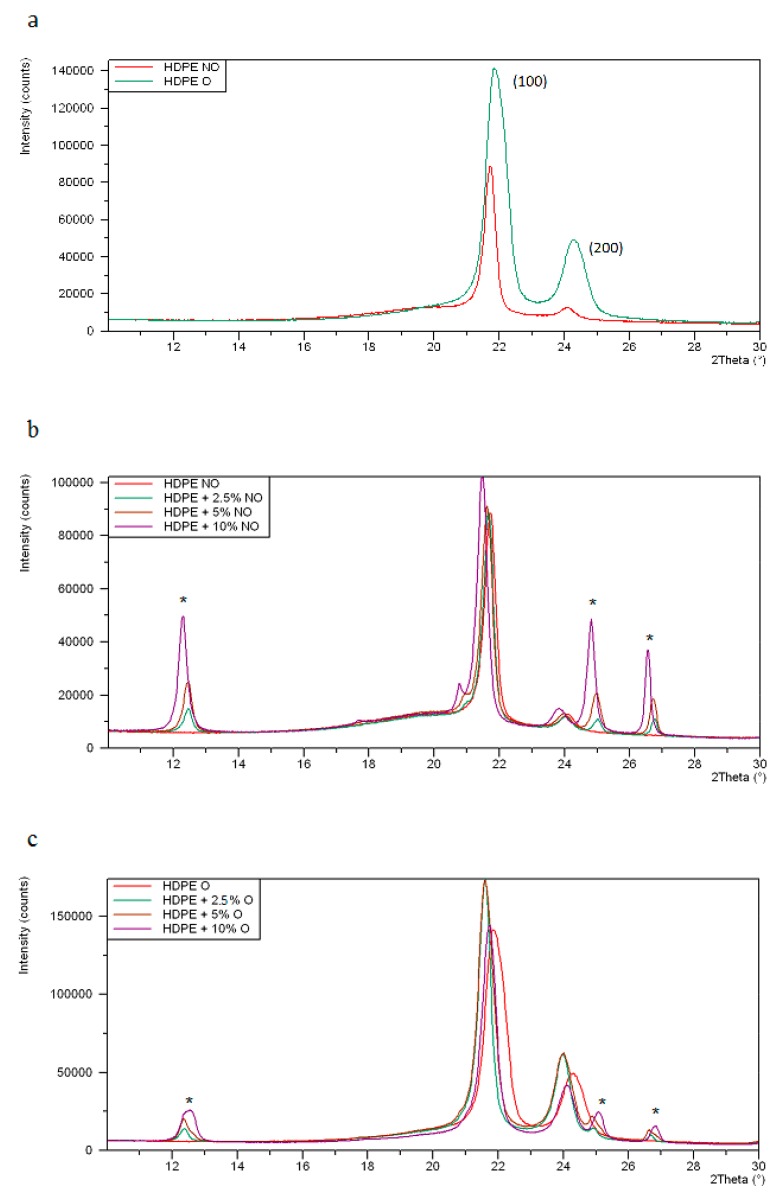
XRD patterns of HDPE (**a**), and its composites with 2.5%, 5% and 10% filler: non-oriented (NO) (**b**) and oriented (O) (**c**). The asterisks mark the signals from the filler.

**Figure 5:**
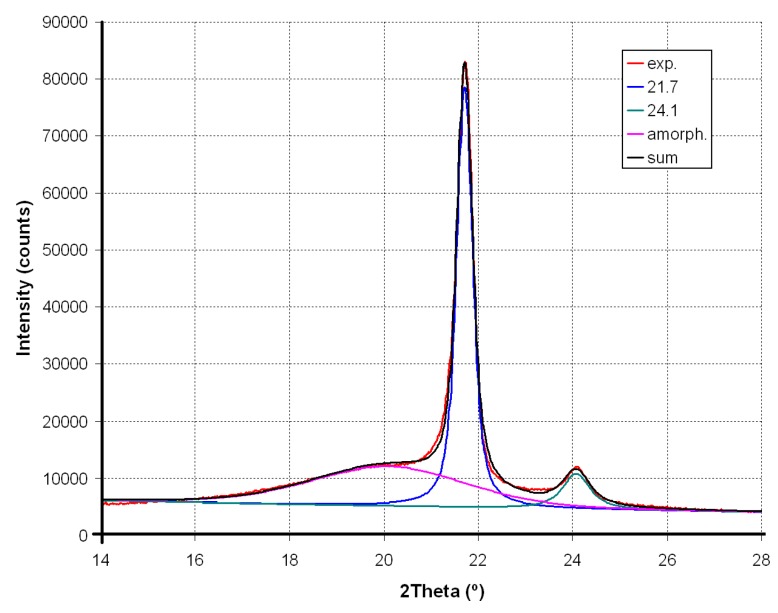
Deconvolution of XRD patterns of HDPE: Red, experimental curve; pink, amorphous halo; blue and green, signals at 2θ = 21.7° (100) and 24.1° (200); black, fitted curve (sum).

The XRD pattern of neat polyethylenes exhibits two main signals at 2θ equal to 21.7° and 24° (Figure 4). Sillikolloid P87 filler is a crystalline substance (main peaks in the 10–30° range of 2θ occur at 12.33°; 20.84°; 24.91° and 26.61°; this last peak is characterized by the highest intensity). In order to calculate the degree of crystallinity (X, %) of PE, deconvolution of experimental XRD curves into components was performed. An exemplary XRD deconvolution for HDPE is shown in Figure 5.

In XRD patterns of HDPE and MDPE composites, the signals attributed to Sillikolloid P87 are also seen and their intensities increase with the increase of its content in the sample.

Unmodified HDPE is characterized by a slightly higher degree of crystallinity than for neat MDPE (Table 1). Sillikolloid P87 added to HDPE reduces its X value by about 6–7%. 

Composites based on the second polymer (MDPE) also show a drop in the degree of crystallinity in the presence of Sillikolloid P87 by several percent (4–11%). This means that the particles of the added filler disturb the order of the macromolecules.

The position of the peaks (2θ, °) in the deconvoluted XRD of the samples with the addition of filler practically does not change. However, the full width at half maximum (FWHM, °) decreases slightly, which may indicate changes in the size of crystallites. According to the Sherrer equation, the FWHM is inversely proportional to the size of the crystallites [40], thus the addition of filler to PE leads to a slight increase of lamellae size in both non-oriented and oriented specimens. As was reported [41], the thicker lamellae decrease the current conductivity in PE insulators. Acting like charge traps, they can therefore have a significant impact on piezoelectric properties.

Stretching in a ratio of 3:1 induces a significant increase in the degree of crystallinity in all tested samples (Table 1). In neat HDPE, X value increases by about 14%, and in MDPE by 23%. This is due to the orientation of the chains in a direction parallel to the direction of stretching, which helps to increase the ordered lamellar areas.

This increase in *X* value for HDPE composites with filler is around 22–30%, and in analogous samples, MDPE is even larger (28–35%). As in the non-oriented samples, the addition of the modifier does not cause the signal shift but the decrease of their half widths (Table 1). This reduction in FWHM due to the presence of the filler (compared to the corresponding value for pure PE) is greater than in the case of non-oriented samples.

### 3.3. Mechanical Properties

Stretching tests have shown that HDPE has higher mechanical resistance than MDPE, which is due to the higher crystallinity of HDPE than MDPE. This is proved by higher values of Young modulus, maximum and breaking stress, as well as corresponding elongations (Table 2). Modification of HDPE by Sillikolloid P87 leads to a decrease of all measured parameters except for the Young module, which increases by approximately 4–12%. 

However, the MDPE composites behaved differently. After filler addition, the improvement of stress at break (σ_B_) and maximal stress (σ_M_), as well as elongation (ε_M_, ε_B_), is observed but Young modulus decreases.

There is no simple, proportional dependence of mechanical properties on the content of filler introduced. A different trend found in composites HDPE and MDPE in the presence of the same modifier results from differences in the structure and order of macromolecules.

Orientation of extruded films in the stretching process (always at the same stretch ratio of 3:1) causes a significant increase in mechanical strength (σ_M_, σ_B_) and Young modulus (Table 2). At the same time, both parameters characterizing elongation (ε_M_, ε_B_) are significantly reduced. This applies to both HDPE and MDPE based systems.

As described in the literature [42], during orientation of semicrystalline PE, the isotropic crystallities (spherulites, axialites, lamella stacks) are transformed into fibrillar structures, which depends on the drawing conditions and chemical structure of macromolecules. In the case of composites, the type and size of filler particles also affect the formation of fibrils. Such crystalline phase transformation can induce a piezoelectric effect.

Interestingly, when comparing oriented samples of neat polymer with oriented samples of composites, one can notice a positive effect of the filler on the tested parameters e.g., on σ_M_ or E_t_ (with some exceptions). 

### 3.4. Thermal Analysis

**Figure 6:**
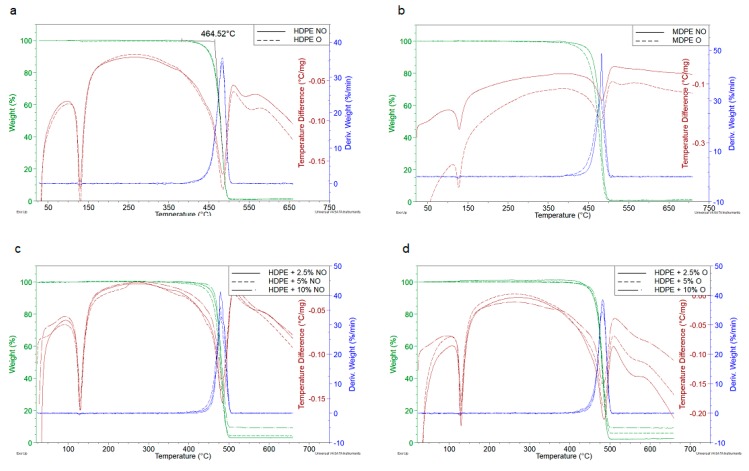
TG, DTG and DTA curves of PO and their composites: Effect of orientation on neat HDPE (**a**) and MDPE (**b**); non-oriented (NO) HDPE+Sillikolloid P87 (**c**), oriented (O) HDPE+Sillikolloid P87 (**d**).

Thermogravimetric analysis showed high thermal stability of all tested samples (Table 3). Selected TG, DTG and DTA curves are shown in Figure 6. 

Figure 6a,b present the comparison of thermogravimetric results for non-oriented and oriented polyethylenes, while Figure 6c,d show the effect of Sillikolloid P87 filler on HDPE. The temperature of the degradation onset, *T*_o_, was determined from the intersection of tangents to the TG curve, while the temperature at maximum process rate was read from DTG, as illustrated in Figure 6a.

The decomposition onset (*T*_o_) is observed at temperatures over 460 °C and the temperature at the maximum process rate (*T*_max_) occurs around 480 °C (Table 3). HDPE begins to decompose at slightly lower temperatures than MDPE but the rate of degradation is clearly higher in the case of MDPE.

The thermal degradation of PE begins with the cracking of weak chemical bonds, which are structural defects in macromolecules (e.g., branching or peroxide groups originating from the polymerization process and forming during processing at elevated temperature). Formed free radicals such as H, OH or alkyl radicals participate in secondary reactions with macromolecules (abstraction of hydrogen atoms and side groups) leading to next macroradicals. Propagation of thermal degradation depends on the rate of diffusion of radicals. The most active species are the small, very mobile radicals but with a temperature increase, the activation energy of the decay of the remaining stable chemical bonds is exceeded, and the rate of decomposition reaches its maximum. The revised thermal degradation mechanism of PE has been recently published by Bracco and coworkers [43].

The polymer decomposition under the tested conditions is one-stage and complete. In neat polymers, the loss of mass is 99–100% at 500 °C. In composites is smaller by several percent, which corresponds to the mineral residue of the introduced filler. DTA curves exhibit two endo-thermic transformations, the first of them is the PE melting peak (which is not accompanied by mass change), the second one corresponds to the thermal destruction, also shown in the TG and DTG curves.

Detailed analysis of parameters determined from TGA curves indicates a slight increase of *T*_o_ in non-oriented HDPE samples under the influence of Sillikolloid P87. MDPE samples behave differently—in this case filler decreases *T*_o_ and *T*_max_. 

Orientation leads to a small increase of *T*_o_ in HDPE (about 2 °C), while this parameter in oriented MDPE decreases by 12 degrees. Changes in oriented samples with fillers are irregular and very slight. Melting points (*T*_m_) also remain unchanged in the samples of different composition. This means that the observed changes in the degree of crystallinity of different samples (found using XRD) have no significant effect on thermal degradation of PE under dynamic conditions and an inert atmosphere.

### 3.5. Piezoelectric Properties

The value of piezoelectric charge of polarized films of PE and its composites was determined at 100 kPa. 

Directly after polarization, non-oriented HDPE film practically does not exhibit piezoelectric charge. The oriented HDPE receives a charge of 285 pC/cm^2^, but unfortunately it decreases significantly after some days of storage.

The non-oriented MDPE film without any additives has a charge of opposite value to the film polarization direction. The value falls by half during one month and then still diminishes. Similarly, the oriented MDPE acquires low charge which decreases over time. Therefore, pure polymer (HDPE and MDPE) in this unmodified form, is not suitable as piezoelectric material.

**Figure 7:**
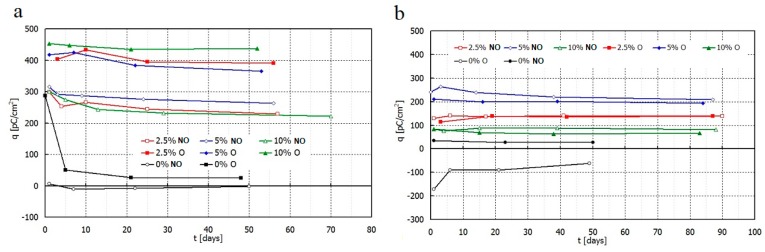
Dependence of piezoelectric charge vs. storing time for non-oriented (NO) and oriented (O) films HDPE+Sillikolloid P87 (**a**) and MDPE+Sillikolloid P87 (**b**) at room temperature.

**Figure 8:**
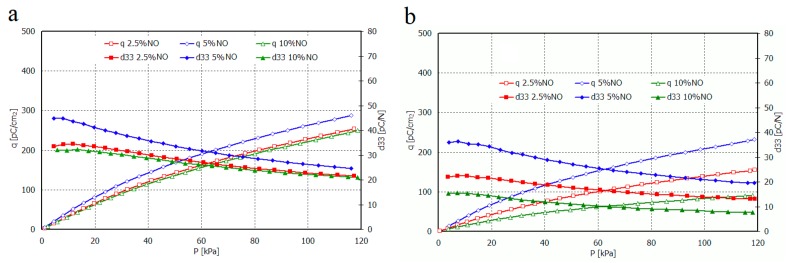
Dependence of piezoelectric charge and piezoelectric coefficient d_33_ vs. mechanical stress for non-oriented (NO) films HDPE+Sillikolloid P87 (**a**) and MDPE+Sillikolloid P87 (**b**).

**Figure 9:**
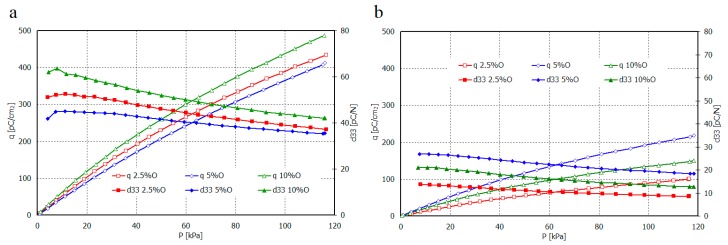
Dependence of piezoelectric charge and piezoelectric coefficient d_33_ vs. mechanical stress for oriented (O) films HDPE+Sillikolloid P87 (**a**) and MDPE+Sillikolloid P87 (**b**).

**Figure 10:**
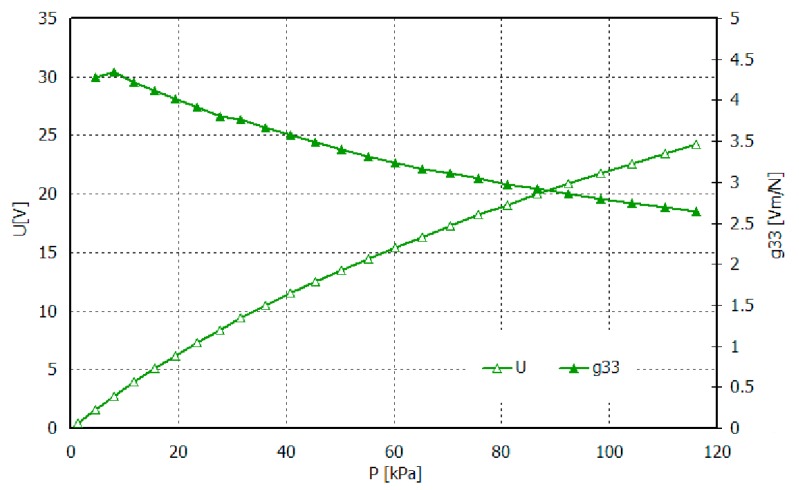
Dependence of tension and piezoelectric coefficient g_33_ vs. mechanical stress for composite HDPE+10% Sillikolloid P87.

PE composites with the addition of filler behave differently. The measurements indicate that samples based on HDPE, MDPE and Sillikolloid P87 received the good piezoelectric properties (Figure 7, Figure 8, Figure 9 and Figure 10). The data from Figure 7 show that both types of composites stabilize ~15 days after the polarization process, in other words, no significant changes in *q* and *U* values are observed within 2–3 months after this time. Filled HDPE exhibit higher values of piezoelectric charge and voltage compared to MDPE with analogous content of Sillikolloid P87.

Orientation of HDPE films causes significant increase of piezoelectric effect in contrast to MDPE composites. In the case of MDPE+Sillikolloid P87 samples, stretching leads to the worsening of the piezo-effect but the changes are insignificant (Figure 7b). Only one composition of MDPE (with 5% filler, non-oriented) showed similar charge to that in non-oriented HDPE composites.

Relation of piezoelectric charge and piezoelectric coefficient d_33_ for non-oriented and oriented films of HDPE+Sillikolloid P87 and MDPE+Sillikolloid P87 from mechanical stress are shown in Figure 8 and Figure 9, respectively. 

The value of piezoelectric coefficient d_33_ for non-oriented samples of both polyethylene types is the greatest for 5 wt.% of the filler in the composite. It attains from ~47 pC/N to ~23 pC/N and from ~35 pC/N to ~20 pC/N, for smaller and greater stresses, respectively. For composite films HDPE+Sillikolloid P87 within greater stresses, d_33_ value is almost similar, independent from filler content. It is probably connected with the optimal distribution of filler particles in polymer matrix. In the case of 10% filler content, the larger aggregates can be formed, which affects both the deterioration of mechanical and piezoelectric properties. The situation changes in these samples after stretching, which is certainly related to the rearrangement of filler particles between parallel oriented PE macrochains.

The greatest value of the d_33_ parameter was obtained for oriented HDPE+10% Sillikolloid P87. It attains ~65 pC/N and 42 pC/N for smaller and greater stresses, respectively. The d_33_ for MDPE+Sillikolloid P87 films is considerably lower; the greatest values are from ~25 pC/N to ~18 pC/N for films with 5 wt. % filler in the matrix. MDPE film orientation caused diminishing of the d_33_ value.

For films of greatest d_33_ values, i.e., oriented HDPE+10% Sillikolloid P87, the g_33_ coefficient was also calculated. The thickness of this film was 79 µm and contact surface, 10 cm^2^. The results are shown in Figure 10. As we can see, the sample of surface 10 cm^2^ may generate an electric voltage up to ~25 V, i.e., under stress perpendicular to the sample plain, consistent to the electric field in the electret. 

## 4. Discussion

The creation of electrets in polymeric composite is associated with the appropriate ordering of macromolecules and the morphology of the samples, which has been emphasized many times in the literature on piezoelectrics [11,12,13,14,15]. The presence of voids in the filled PE, observed by SEM, allow for accumulation of electric charge at the interface in this nonpolar polymeric matrix. Such a voided structure, also called cellular structure, has been described in literature for filled polypropylene [29,32,33] or foamed PE [34] and for PVDF [44] exhibiting high piezoelectric effect. 

As XRD research has shown, the degree of crystallinity of both PE’s increases significantly after the orientation process, which has an impact on the improvement of mechanical properties. However, these are not the only criteria for obtaining electrets. Also, the type of polymer, subtle differences in chemical structure, as well as added processing aids play a big role here. 

Both types of PE are copolymers of ethylene and hexene-1, they contain antioxidants and acid scavenger but the detail chemical composition is not given by the manufacturer. HDPE and MDPE differ in density (0.946 and 0.938 g/cm^3^ for HDPE and MDPE, respectively) and other properties, which are given in the technical data sheets [35,36].

As it is known from the literature, PE properties depend on the polymerization method and conditions and the type of catalyst used [45,46]. The microstructure of PE depends on the number and length of branching, as well as the type of end groups in carbon chains. Generally, the stronger interactions between regular chains are in HDPE, whereas in a PE of lower density resulting from more branching, these interactions are weaker and such a polymer becomes more susceptible to deformation. In pure polyolefins these are only dispersive interactions, however, in the presence of aluminosilicate particles, dipole type interactions also occur. As can be seen from the above studies, it also affects the piezoelectric properties.

**Figure 11:**
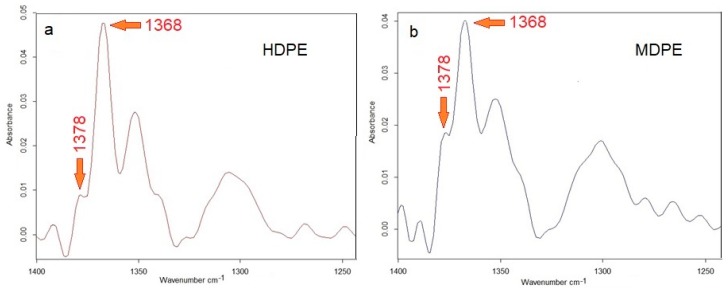
Comparison of FTIR spectra in the range of 1200–1400 cm^−1^ for HDPE (**a**) and MDPE (**b**); 1378 and 1368 cm^−1^ bands correspond to symmetric scissoring vibrations of CH_3_ and CH_2_, respectively.

Unexpected differences in piezoelectric properties of HDPE and MDPE-based composites can be explained by slight differences in the structure of both polymers which can be detected on the basis of a precise FTIR spectroscopic analysis [45]. Figure 11 shows the FTIR spectra in the range corresponding to the deformation vibrations of the CH_3_ (1378 cm^−1^) and CH_2_ (1368 cm^−1^) groups. The lower intensity of the peak at 1378 cm^−1^ corresponding end-CH_3_ groups (Figure 11a) clearly indicates a lower branching degree in HDPE compared to MDPE (Figure 11b). This is confirmed by higher density, a higher degree of crystallinity and is also reflected in the higher Young’s module of HDPE than those parameters in MDPE.

Finally, in order to estimate the extent of the piezoelectric response and the suitability of the tested systems for practical applications, the obtained piezoelectric coefficients can be compared with the corresponding values for poly(vinylidene fluoride) (PVDF), which is regarded as a best piezoelectric material among polymers. According to literature reports, d_33_ of PVDF ranges from over a dozen to about 34 pC/N [14,22,47]. HDPE composites presented above exhibit even higher d_33_ values, which is a very promising result.

## 5. Conclusions

The experimental part showed the possibility of manufacturing piezoelectric composites on the base of polyethylene with aluminosilicate filler. The determined d_33_ coefficients for PE composites are even larger than for PVDF, which may be a reference to the comparison of piezoelectric properties. 

HDPE+Sillikolloid P87 generates and stores more electric charge than MDPE+Sillikolloid P87, which results from subtle differences in the chemical structure of both polymers and the morphology of the composites produced. 

Thermogravimetric studies provided additional information on the properties of composites and showed that they are resistant to high temperatures, which broadens the possibilities of their potential application and the possibility of forming any shapes from the melt state. The tested composites are also characterized by good mechanical strength and a high Young’s modulus. Therefore, one can recommend these materials for the production of cheap, flexible piezoelectric sensors (including wearable devices equipped with personal sensors), actuators or energy harvesters for general use. 

Finally, we can state that the selected instrumental techniques allow for the proper characterization of the proposed new piezoelectric materials, enabling the optimal composition from the point of view of practical requirements to be determined. It was found that, the ordered cellular structure of the HDPE matrix with 5% filler additive ensures very good piezoelectric properties. 

It should be pointed out that in order to obtain reproducible piezoelectric materials based on polyethylene composites, a strict regime of their manufacture must be respected.

## Figures and Tables

**Table 1:** Results of XRD analysis for non-oriented (NO) and oriented (O) HDPE, MDPE and their composites with Sillikolloid filler (FWHM, full width at half maximum of peak; *X*, crystallinity degree of PE).

Sample (PE Type + Filler Content, %)		Amorphous Halo Share, %	Position (2θ, °), FWHM (°) and Share (%) of PE Crystalline Signals						*X*, %
			1			2			
			2θ	FWHM	%	2θ	FWHM	%	
HDPE	NO	42.6	21.7	0.40	50.3	24.1	0.51	7.1	57.4
HDPE+2.5	NO	49.2	21.7	0.36	46.4	24.0	0.45	4.4	50.8
HDPE+5	NO	51.0	21.6	0.39	44.3	24.0	0.44	4.7	49.0
HDPE+10	NO	48.5	21.5	0.37	45.5	23.8	0.43	6.0	51.5
HDPE	O	28.7	21.9	0.71	50.7	24.3	0.76	20.6	71.3
HDPE+2.5	O	23.1	21.6	0.43	54.0	24.0	0.52	22.8	76.9
HDPE+5	O	20.3	21.6	0.54	57.2	24.0	0.58	22.5	79.7
HDPE+10	O	24.9	21.7	0.50	56.2	24.1	0.54	18.8	75.1
MDPE	NO	47.8	21.7	0.42	47.7	24.0	0.54	4.6	52.2
MDPE+2.5	NO	57.9	21.6	0.41	38.3	23.9	0.53	3.9	42.1
MDPE+5	NO	58.7	21.5	0.41	37.2	23.9	0.48	4.1	41.2
MDPE+10	NO	54.9	21.5	0.40	41.7	23.8	0.45	3.5	45.1
MDPE	O	24.6	22.0	0.56	58.2	24.3	0.65	17.2	75.4
MDPE+2.5	O	24.2	21.6	0.42	57.9	24.0	0.51	18.0	75.8
MDPE+5	O	22.7	21.6	0.52	59.8	24.0	0.58	17.5	77.3
MDPE+10	O	24.1	21.8	0.48	58.9	24.1	0.54	17.0	75.9

**Table 2:** Mechanical parameters of non-oriented (NO) and oriented (O) HDPE, MDPE and their composites with Sillikolloid (σ_M_, maximal stress, σ_B_, stress at break, ε_M_, maximal elongation, ε_B_, elongation at break, E_t_, Young modulus).

Sample (PE Type +Filler Content, %)		σ_M_, MPa	σ_B_, MPa	ε_M_, %	ε_B_, %	*E*_t_, MPa
HDPE	NO	36	36	694	695	596
HDPE+2.5	NO	28	28	631	632	643
HDPE+5	NO	29	29	604	604	669
HDPE+10	NO	31	31	556	557	618
HDPE	O	112	30	113	124	949
HDPE+2.5	O	103	103	140	140	868
HDPE+5	O	115	114	125	126	1166
HDPE+10	O	110	118	78	97	1233
MDPE	NO	27	26	680	681	421
MDPE+2.5	NO	38	38	743	743	407
MDPE+5	NO	44	44	799	800	388
MDPE+10	NO	47	47	793	795	414
MDPE	O	107	107	148	148	746
MDPE+2.5	O	120	90	94	100	838
MDPE+5	O	117	53	80	90	790
MDPE+10	O	124	49	76	83	884

**Table 3:** Thermal parameters for non-oriented (NO) and oriented (O) HDPE, MDPE and their composites with Sillikolloid (*T*_o_, temperature onset, *T*_max_, temperature at maximum process rate, ∆*m*, weight loss at 600 °C, *T*_m_, melting temperature, *V*_max_, maximal rate of decomposition).

Sample (PE Type + Filler Content, %)		*T*_o_, °C	*T*_max_, °C	∆*m*, %	*T*_m_, °C	*V*_max_, %/min
HDPE	NO	464	483	99	129	35
HDPE+2.5	NO	468	480	97	130	39
HDPE+5	NO	464	478	96	130	36
HDPE+10	NO	468	481	90	130	33
HDPE	O	466	483	98	130	36
HDPE+2.5	O	466	482	98	129	39
HDPE+5	O	467	480	94	128	37
HDPE+10	O	467	481	90	130	35
MDPE	NO	470	482	99	128	48
MDPE+2.5	NO	465	478	98	126	36
MDPE+5	NO	468	479	94	126	41
MDPE+10	NO	465	478	93	126	34
MDPE	O	458	478	100	127	31
MDPE+2.5	O	465	481	97	127	36
MDPE+5	O	465	482	96	125	37
MDPE+10	O	462	481	86	126	29
